# Association of single nucleotide polymorphic sites in candidate genes with aggressiveness and deoxynivalenol production in *Fusarium graminearum *causing wheat head blight

**DOI:** 10.1186/1471-2156-13-14

**Published:** 2012-03-12

**Authors:** Firas Talas, Tobias Würschum, Jochen C Reif, Heiko K Parzies, Thomas Miedaner

**Affiliations:** 1 Universitaet Hohenheim, State Plant Breeding Institute (720), Fruwirthstr. 21, 70593 Stuttgart, Germany; 2 Universitaet Hohenheim (350), Institute of Plant Breeding, Seed Science & Population Genetics, Fruwirthstr. 21, 70593 Stuttgart, Germany; 3 National Commission of Biotechnology (NCBT), P. O. Box. 31902, Damascus, Syria

**Keywords:** Aggressiveness, Association mapping, DON, *Fusarium graminearum*, FHB, Linkage disequilibrium, QTL, *Triticum aestivum*, SNP

## Abstract

**Background:**

*Fusarium graminearum *sensu stricto (s.s.) is an ubiquitous pathogen of cereals. The economic impact of Fusarium head blight (FHB) is characterized by crop losses and mycotoxin contamination. Our objective was to associate SNP diversity within candidate genes with phenotypic traits. A total of 77 *F. graminearum *s.s. isolates was tested for severity of fungal infection (= aggressiveness) and deoxynivalenol (DON) production in an inoculated field experiment at two locations in each of two years. For seven genes known to control fungal growth (*MetAP1, Erf2*) or DON production (*TRI1, TRI5, TRI6 TRI10 *and *TRI14*) single nucleotides polymorphic sites (SNPs) were determined and evaluated for the extent of linkage disequilibrium (LD). Associations of SNPs with both phenotypic traits were tested using linear mixed models.

**Results:**

Decay of LD was in most instances fast. Two neighboring SNPs in *MetAP1 *and one SNP in *Erf2 *were significantly (*P *< 0.05) associated with aggressiveness explaining proportions of genotypic variance (*p_G_*) of 25.6%, 0.5%, and 13.1%, respectively. One SNP in *TRI1 *was significantly associated with DON production (*p_G _*= 4.4).

**Conclusions:**

We argue that using the published sequence information of *Fusarium graminearum *as a template to amplify comparative sequence parts of candidate genes is an effective method to detect quantitative trait loci. Our findings underline the potential of candidate gene association mapping approaches to identify functional SNPs underlying aggressiveness and DON production for *F. graminearum *s.s populations.

## Background

Fusarium head blight (FHB) is a destructive disease to cereals including wheat and barley. *Fusarium graminearum *(teleomorph *Gibberella zeae*) is considered to be the main causal agent of this disease in addition to other species such as *F. culmorum *[1]. *F. graminearum *complex has been subdivided into several cryptic species [2], the main member in Germany is *F. graminearum *sensu stricto (s.s.) [3]. FHB disease leads to prematurely bleached spikes in infected plants [1] with considerable yield losses and contamination by mycotoxins such as deoxynivalenol (DON) [4]. DON is the most common food and feed contaminant in Europe and the maximum permissible level in unprocessed wheat used for food is 1.25 mg kg^-1 ^[5].

The quantitative ability of an isolate to cause disease on a susceptible host plant in a non-race specific pathosystem is defined as aggressiveness [6]. Aggressiveness is an important factor determining the potential ability of an isolate to cause yield losses. Large genetic variation of aggressiveness, type of mycotoxin, and DON production was found among isolates sampled in the same country or even the same field [7-11]. The molecular causes of this tremendous variation in aggressiveness and DON production in *F. graminearum *are still unclear. To date, only one QTL (quantitative trait locus) study from a single cross was reported detecting at least two QTL for aggressiveness [12]. It was shown before, that a large amount of molecular variation was found within field populations of *F. graminearum *s.s. using SSR (simple sequence repeat) markers. Comparing 12 individual field populations each consisting of 30 isolates, Talas et al. [3] reported that 71% of the molecular variation was assigned within populations and 29% between populations. A cause for this high diversity might be the mating system. *F. graminearum *is a haploid, homothallic fungus and propagates asexually by conidia and sexually by ascospores allowing both selfing and outcrossing [7]. With the availability of the full genomic sequence of *F. graminearum *[13], it is now possible to deeper analyze this variation by a candidate gene approach. An array of candidate genes, including components of transcription, signal transduction, host-specific nutrition, host infection/colonization and trichothecene biosynthesis is available in the version 3.2 of the Pathogen-Host Interactions database [14]. The candidate genes we were aiming for are known to control DON biosynthesis and/or aggressiveness (*TRI1, TRI5, TRI6, TRI10, TRI14*) [15,16] or are expected to have a link to aggressiveness but are yet uncharacterized for *Fusarium *spp. (*MetAP1, Erf2*) [17,18]. Candidate gene association mapping is a sensitive tool if the mapping resolution is high enough [19]. The resolution of association mapping depends on the extent of linkage disequilibrium (LD), i.e. the non-random association of alleles present in a species [20]. Low LD was reported in *F. graminearum *estimated by different types of genetic makers such as VNTR, AFLP, RFLP [7,11,21], but no data on LD within and among genes is available. Because LD is species and population specific, it should consequently be determined before conducting an association mapping study.

The specific objectives of our study were to: (i) investigate the nucleotide diversity on gene level, (ii) investigate the extent of LD between single nucleotide polymorphic sites (SNPs) within and among candidate genes, (iii) identify SNPs of candidate genes *Erf2 *and *MetAP1 *for testing whether they affect the quantitative variation for aggressiveness, and (iv) evaluate associations of SNPs in *TRI1 *and other four genes of the *TRI5 *cluster with variation in DON production.

## Results

### Phenotypic analysis

Phenotypic data were analyzed in detail in a companion study [9]. All 77 *F. graminearum *s.s. isolates produced symptoms of FHB disease in all environments. Briefly, genotypic proportion of phenotypic variance of the isolates was significant (*P *< 0.01) for both traits: Aggressiveness measured as FHB rating on scale from 0 to 100 and DON production measured as DON concentration in wheat kernels in mg kg^-1^. Isolate × environment interaction variance was significant (*P *< 0.01) only for mean FHB rating but not for DON production. Entry-mean heritability was moderate for mean FHB rating (0.55) and DON production (0.62).

**Figure 1:**
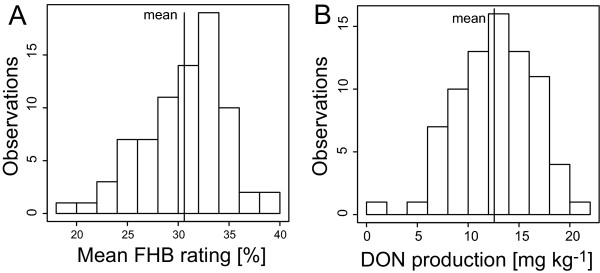
**Histograms for 77 *F. graminearum* isolates. **Best linear unbiased estimates (BLUEs) for mean FHB rating (A) and DON content (B) calculated across four environments (location × year combinations)

Histograms based on best linear unbiased estimators (BLUEs) followed a normal distribution for both traits (Figure 1). The mean of FHB infection among all isolates was 30.6% ranging from 18.4 to 38.9%. Average DON production was 12.6 mg kg^-1 ^ranging from 3.5 to 21.6 mg kg^-1^. One isolate (WET24) produced a very low concentration of DON (0.3 mg kg^-1^) and was found to be a nivalenol producer by chemotype-specific primers.

### Analysis of population structure and diversity

**Figure 2:**
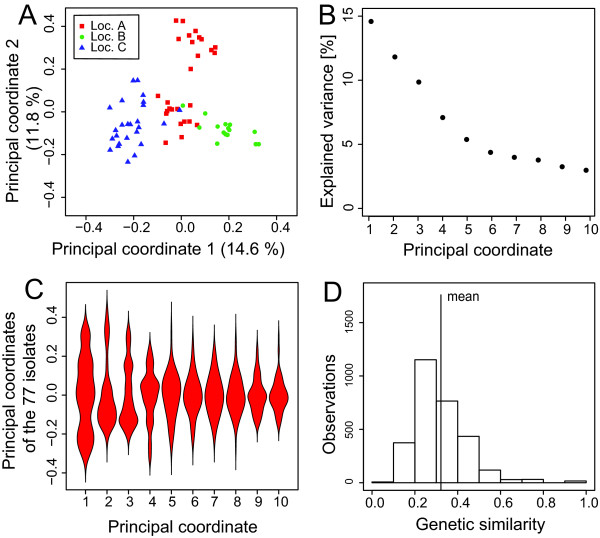
**Population structure and familial relatedness**. (**A**) Principal coordinate analysis of the 77 isolates, based on modified Rogers' distance estimates whereas location A = Hohenheim, location B = Schickelsheim, and location C = Wetze, (**B**) Explained genotypic variance of the first ten principal coordinates, (**C**) Violin plot showing the density distribution of the first ten principal coordinates, (**D**) Histogram of the genetic similarities among the 77 isolates.

Principal coordinate analysis (PCoA) based on modified Rogers' distances between all isolates did not show a distinct separation of the isolates sampled from different locations (Figure 2A). Explained variance gradually decreased according to the first ten principal coordinates (Figure 2B). The violin plot (Figure 2C) had a continuous density of distribution over all ten principal coordinates without any division within the principal coordinates. Genetic similarity ranged from 0.057 to 1.0 with a mean value of 0.31 (Figure 2D).

### Estimation of nucleotide diversity and linkage disequilibrium

**Figure 3:**
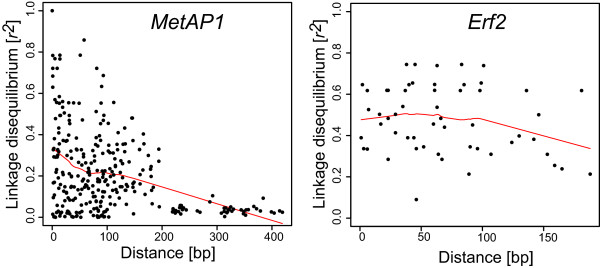
**Linkage disequilibrium (LD) decay**. Robust locally fitted regression of linkage disequilibrium over base-pair distance within the two candidate genes, *MetAP1 *(25 SNPs) and *Erf2 *(11 SNPs).

**Figure 4:**
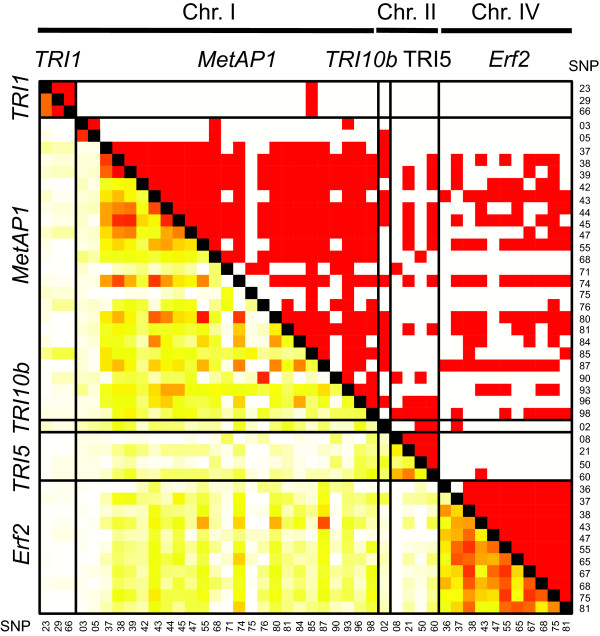
**Pairwaise linkage disequilibrium (LD)**. The structure of LD within and among five candidate genes for 77 *Fusarium *isolates. Significant LD (*P *< 0.05, above diagonal) and LD measured as *r^2 ^*between all pairs of selected SNP loci (below diagonal). The horizontal and vertical lines separate the candidate genes, red coloring indicates significant LD and the higher the *r^2 ^*values (*r^2 ^*≥ 0.1), the darker is the used color, white indicates non-significant LD or *r^2 ^*= 0.

Percentage of polymorphic sites per total sequenced region of each gene (without singletons) varied from 0.9% (5/513) on *TRI10b *to 8.8% (65/734) on *MetAP1 *indicating a high nucleotide diversity in most tested genes (Table 1). LD of SNPs within the gene *MetAP1 *decayed rapidly within 200 bp of physical distance, i.e., the robust locally fitted regression of *r^2 ^*values has a trend to decay from *r^2 ^*= 0.35 to *r^2 ^*< 0.1, whereas LD within the gene *Erf2 *had *r^2 ^*values ranging from 0.8 to 0.2 and the regression of *r^2 ^*trends to decay already after 150 bp (Figure 3). LD with *r^2 ^*values higher than 0.1 were detected between all allele combinations within the tested genes followed by a rapid decay negatively correlated with the physical distance in base pair (Figure 4). In line with the rapid decay, LD between genes located on the same chromosome (i.e., *TRI1 *and *MetAP1*) is low (*r^2 ^*< 0.1). Interestingly, 48%, 19%, and 45% of the SNP pairs between the genes *TRI10*/*MetAP1, MetAP1*/*TRI5 *and *MetAP1*/*Erf2*, respectively, have higher values of *r^2 ^*than 0.1, although they are located on different chromosomes. Low *r^2 ^*values (< 0.1) were observed for SNPs of gene pairs *TRI10*/*Erf2 *and *TRI5*/*Erf2*, whereas *r^2 ^*values of 0.2 were detected between SNP pairs of *TRI10b *and *TRI5*, followed by a rapid decay of LD.

**Table 1:** Sequenced regions of candidate genes and its related nucleotide polymorphism

Gene ID^a^	Sequenced region relative to the ATG	No. of SNPs detected	No. of singletons	Nucleotide diversity (%)^b^	No. of SNPs with allele frequency > 0.1
*TRI1 *(FGSG_00071)	311-1065	73	54	2.5	3
*TRI6 *(FGSG_16251)	35-558	36	29	1.3	0
*TRI5 *(FGSG_03537)	479-1080	70	38	5.3	4
*TRI10 *a (FGSG_03538)	31-678	123	80	6.6	0
*TRI10 *b (FGSG_03538)	760-1273	34	29	0.9	1
*TRI14 *(FGSG_03543)	277-976	43	23	2.9	0
*MetAP1 *(FGSG_01397)	200-934	106	41	8.8	25
*Erf2 *(FGSG_08531)	1193-1825	80	42	6.0	11

^a ^The given gene ID is the entry number of the MIPS *F. graminearum *genome database (FGDB; Wong et al. 2011)

^b ^Nucleotide diversity is the frequency of SNPs (without singletons) relative to the total length of the sequenced gene region

### Association analysis for aggressiveness and mycotoxin

**Figure 5:**
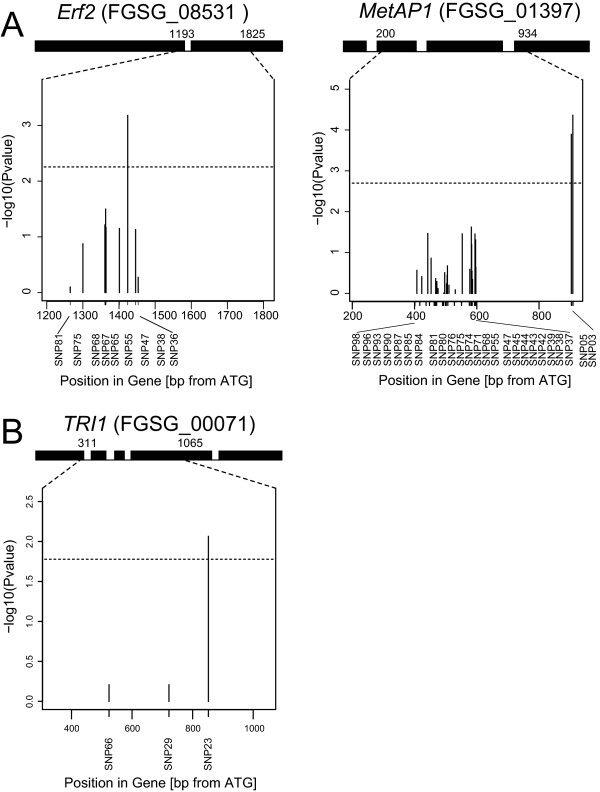
**Significant associations in the candidate genes**. (**A**) *Erf2 *and *MetAp1 *for mean FHB rating and (**B**) *TRI1 *for DON content. The dashed horizontal line indicates the threshold (Bonferroni-corrected *P *< 0.05).

Two adjacent SNPs of the gene *MetAP1 *were significantly associated (*P *< 0.05 using Bonferroni-Holm correction) [22] with mean FHB rating with an explained genotypic proportion of variance *p_G _*= 25.6 and 0.5, respectively (Table 2, Figure 5A). Additionally, one SNP in the gene *Erf2 *was significantly associated with this trait showing a *p_G _*of 13.1% (Table 2, Figure 5A). A single SNP significantly associated with DON production was identified in the *TRI1 *gene explaining 4.4% of the genotypic variance of DON production (Table 2, Figure 5B). All detected SNPs that were associated to the mentioned phenotypic traits were non-synonymously substituted (Table 2). Applying a haplotype analysis across genes, we could confirm a significant association between mean FHB rating and *MetAP1 *and between DON production and *TRI1* (Additional file1).

**Table 2:** SNPs in the candidate genes significantly associated with mean FHB rating or DON content

Trait/Candidate gene	SNP #	Position^a^	Polymorphism	Changes in amino acids^b^	*P*-value	*p_G _*(%)^c^
Mean FHB rating (%)						
*MetAP1*	SNP 05	904	A, G	H, R	1.28 × 10^-4^	25.6
	SNP 03	909	A, C	T, P	1.94 × 10^-4^	0.5
*Erf2*	SNP 47	1424	C, T	Q, Stop	6.61 × 10^-4^	13.1
DON production (mg kg^-1^)						
*TRI1*	SNP 23	851	A, G	R, Q	8.62 × 10^-3^	4.4

^a ^The position is relative to the start codon (ATG)

^b ^Different amino acids coded by SNPs located in exon regions (non-synonymous SNPs)

^c ^The proportion of explained genotypic variance in percent (*p_G_*) after Bonferroni-Holm procedure

## Discussion

Association mapping based on candidate genes is a promising tool for high-resolution mapping of genes contributing to quantitative traits [23]. Nevertheless, it has not yet applied to investigate the basis of quantitative variation in aggressiveness and/or DON production in *F. graminearum*. This is a totally different approach than using knock-out mutants [24], because we are aiming for the analysis of quantitative differences produced by single nucleotide changes of the respective genes in a set of 77 isolates. The high number of singletons may, besides sequencing noise, be caused by frequent sexual recombination of the fungus. Therefore, we used a strict threshold and were analyzing only SNPs with allele frequencies > 0.1.

### Choice of candidate genes

The genes for deoxynivalenol (DON) biosynthesis reside primarily in a 25 kb cluster (*TRI5 *cluster), *TRI1 *gene belong to a second smaller cluster that has a different chromosomal localization [16]. *TRI1, TRI5, TRI14 *have a direct effect on the production of DON or acetylated DON, *TRI6 *and *TRI10 *are regulating this pathway. *TRI14 *export the deoxynivalenol outside the mycelia in addition to its major role in aggressiveness [15].

The function of *MetAP1 *is still not described in *Fusarium *spp., a deletion, however, reduces growth in *Saccharomyces cerevisiae *[18,25,26]. In both, prokaryotes and eukaryotes the N-terminal methionine is often cleaved by methionine aminopeptidase encoded by the gene *MetAP1 *[18]. *Erf2 *gene is a component of the *RAS *protein subcellular localization pathway in yeast [17]. *RAS2 *gene is known to affect the pathogenesis of *F. graminearum *by regulation of hyphal growth and expression of hydrolytic enzymes [27]. Failing of palmitoylation mainly controlled by the *Erf2 *gene in *S. cerevisiae *reduces amount of RAS protein at the plasma membrane and lead to poor growth of yeast [17]. Both genes are not indispensible for fungal growth because their deletion does not prevent any growth at least in *S. cerevisiae*. And the 77 isolates analyzed in this study, all had a similar growth *in vitro*, produced enough spores for inoculation and were able to infect the host in the field. Significant, quantitative differences for aggressiveness and DON production, however, were found illustrating those genes with regulatory functions might be involved. Implementing associations between new candidate genes and important phenotypic traits may provide a useful tool to rapidly check a possible contribution of these genes to QTL of DON production or aggressiveness in *F. graminearum *as shown previously in mammal research [23]. If an association is found, a more detailed analysis of the function of the respective genes should, of course, follow.

### Molecular diversity of the 77 isolates

All isolates were checked by respective primers for their designation to *F. graminearum *s.s. Nucleotide diversity within candidate genes of this study ranged from 1 to 9 per 100 bp being high compared to 0 to 2 SNPs per 100 bp reported by Cuomo et al. [13]. This was not unexpected since Cuomo et al. [13] used just two isolates (PH-1 and GZ3639) while in this study 77 isolates were analyzed. Many genes in *F. graminearum *are localized in regions of high SNP density, i.e. highly variable regions, especially those expressed during plant infection. Moreover, it is known that the density of SNPs is biased in *F. graminearum *in a way that 50% of the SNPs are within 13% of the genome sequence, the highest SNP density was reported in some regions of chromosome II, where most of the *TRI *genes are located [13]. The high nucleotide diversity of these groups of genes suggests that the fungus has a great capacity for adaptability and genetic change during its interaction with the host plant [13]. Despite this, we found much more SNPs with allele frequencies > 0.1 in the *MetAP1 *and *Erf2 *genes than in the *TRI *genes analyzed. This was not the case, when just the number of detected SNPs is regarded, indicating that nucleotide diversity may be similar, but more rare haplotypes occur in the *TRI *genes. Moreover, high SNPs density was detected in one or two large interstitial regions on chomosomes I, II, and IV [13].

### Population structure and consequences for association mapping

Correcting for population and/or family structure is essential for association mapping to decrease the number of false positive QTL [28]. An appropriate statistical model should provide an excellent compromise between correcting for population stratification to decrease the probability of detecting false positive SNP-trait associations but still retaining enough information within the SNPs for QTL detection [29]. Although some grouping according to the sampling location might be seen in the PCoA, the violin plots did not show any separated knots on the first ten principle coordinates (Figure 2). Thus, no distinct subpopulations were identified and correction for familial relatedness between isolates should be sufficient. Therefore, trait-SNP association was investigated in detail with the K model, which incorporated estimates of kinship coefficients based on SSR data.

### Extent of linkage disequilibrium and resolution of association mapping

Rapid decay of regression parameter *r^2 ^*in a short physical distance of 200 bp within two genes was presented in this study (Figure 3), providing a high resolution of association mapping. Generally, low LD was reported in *F. graminearum *populations using selection-neutral markers (VNTR, AFLP, or RFLP) especially in the region that includes the *TRI*5 gene cluster [11]. Moreover, the weak correlation between *r^2 ^*and physical distance in addition to the large proportion of unlinked SNP with significant LD, such as SNPs between gene pairs *TRI5*/*MetAP1, TRI10*/*MetAP1*, or *MetAP1*/*Erf2 *(Figure 4), suggest the presence of other forces generating LD between unlinked SNPs. The values of *r^2 ^*between most pairs of SNPs in *TRI5*/*TRI10b *are > 0.1, presenting these two genes in LD, that might refer to the controlling role of *TRI10 *on *TRI*5 gene cluster [24,30,31]. That *TRI10 *and *TRI5 *are located close to each other on the same chromosome play a minor role hence the detected LD decay is occurring within 200 bp. Selection acting on oligo- or polygenic traits such as aggressiveness and DON production might be responsible for this [32]; an alternative explanation would be the involvement in the same trait network with other physiologically important genes.

### Association mapping of genes underlying aggressiveness and DON production

We identified three SNPs related to aggressiveness and one SNP related to DON production using the fairly conservative Bonferroni-Holm correction. Setting the threshold for detection of trait associations to an allele frequency of 0.1 might underestimate the number of QTL detected but is due to the restricted population size. Association between SNPs of candidate gene *Erf2 *and phenotypic data revealed a significant association to mean FHB rating, but not to DON content. This might be explained by the fact that the *Erf2 *gene is involved in *RAS*2 processing or trafficking that precedes palmitoylation of *RAS2 *genes [17]. *RAS2 *is known to regulate the aggressiveness through affecting fungal growth and regulating other pathogenicity genes, e.g., *Gmpk1*, which controls the induction of extracellular enzymes required for pathogenesis [27]. Further study is needed to understand how *Erf2 *affects differences in aggressiveness of *F. graminearum *isolates.

Two SNPs in *MetAP1 *gene significantly associated with aggressiveness were located adjacent to each other forming a collinearity pattern. The role of *MetAP1 *was reported in *S. cerevisiae *as reducing cell growth by N-terminal protein modification. A similar role might be expected for *MetAP1 *in *F. graminearum*, hence significant positive correlations between aggressiveness and fungal biomass (*r *= 0.7, *P *= 0.01), and fungal biomass and DON content (*r *= 0.8, *P *= 0.01) were reported among 50 *F. graminearum *isolates [33]. Nevertheless, analyzed isolates were sampled from visually diseased spikelets, thus all were able to infect wheat ears and induce symptoms, non-aggressive isolates were not included in our *F. graminearum *population sample.

A single SNP detected in *TRI1 *was associated with DON production in *F. graminearum. TRI1 *encodes a cytochrome P450 monooxygenase that catalyzes hydroxylation of C-8 position during trichothecene biosynthesis [34]. This confirmation change obviously does not affect aggressiveness, because the respective SNP is just associated with DON content but not with aggressiveness.

## Conclusions

This is the first candidate gene association mapping study provided insights on some genes involved in aggressiveness and DON concentration of *F. graminearum *s.s. The described associations should be validated using a larger number of isolates and different environments. The validated genes are an important starting point for further functional analyses.

## Methods

### Fungal material

Ears of winter wheat (*Triticum aestivum *L.) showing symptoms of FHB were sampled from three commercial fields in Germany to establish a fungal population of *F. graminearum *s.s.: Stuttgart-Hohenheim in southwest Germany in 2008, Wetze and Schickelsheim in Lower Saxony in 2006 and 2007, respectively. From each infected head, one isolate was recovered, transferred onto a fresh SNA plate and placed under permanent UV light for induction of sporulation as described recently [3]. From each isolate, one single spore was picked out under the microscope and transferred onto a fresh SNA plate to establish a single-spore culture. The single-spore isolates were checked morphologically and analyzed for their species specificity and chemotype by different PCR-based assays as described recently in detail [3]. In total, 77 single-spore isolates of *F. graminearum *s.s. (former lineage 7) were inoculated on the moderately resistant German spring wheat cultivar Taifun (KWS LOCHOW GMBH, Bergen, Germany) for analyzing aggressiveness and DON production in the field.

### Design of field studies

Field experiments were planted at each of two locations in 2009 and 2010: Hohenheim (HOH, longitude 9° 12' 58″, latitude 48° 42' 50″, altitude 400 m) and Oberer Lindenhof (OLI, longitude 9° 18' 12″, latitude 48° 28' 26″, altitude 700 m), resulting in four environments (location × year combinations). Mean annual temperatures at HOH and OLI were 10.1°C and 9.0°C, respectively, mean annual precipitations were 644 mm and 723 mm, respectively, across 2009 and 2010. Plants were grown in two-rowed micro-plots of 1 m length and 0.42 m width. Plots were arranged in a chessboard-like design, i.e., each plot with a wheat entry was bordered by four plots of similar size that were planted with a long-strawed spring triticale cultivar Nilex (NORDSAAT GmbH, Halberstadt, Germany) to reduce inter-plot interference caused by drifting of inoculum during spraying or secondary distribution of spores. The experiment was arranged in a split-plot design with two replications. The main plot factor was the *F. graminearum *s.s. population, the subplot factor the isolate. Both factors were assigned to a randomized complete block design. Eight plots per replication included in the subplot factor were not inoculated to estimate the degree of natural infection.

Inoculum was sprayed with a concentration of 2 × 10^5 ^spores ml^-1 ^onto the wheat heads of each plot. Inoculation was performed at full flowering time of the wheat cultivar to ensure maximum susceptibility of wheat to *F. graminearum*. Fusarium head blight (FHB) aggressiveness was rated visually four times as the percentage of infected spikelets per plot (0-100%). This rating included both the number of infected spikes per plot and the number of infected spikelets per spike. To compare FHB reactions, the arithmetic means of four ratings were used and assigned as mean FHB rating throughout the paper. All plots were harvested by hand, threshed, and the grain analyzed to quantify the amount of DON by a commercially available immunotest (R-biopharm AG, Darmstadt, Germany) as previously described in detail [9]. This test cannot differentiate between DON and 3-ADON, so the results include both mycotoxins. Generally, however, the amount of 3-ADON is only 2-3% of total DON content [35]. In each of the four environments, the natural infection rate was very low ranging from 0 to 3% FHB rating and from 0 to 0.34 mg kg^-1 ^DON concentration. Prediction of NIV chemotype was done using the primers N11, 15D11, 3D11, and 11R in a multiplex polymerase chain reaction (PCR) as designed by Zhang et al. [36].

### Detection of population structure and gene sequencing

In order to analyze the population structure, all isolates were fingerprinted with 19 simple sequence repeat (SSR) markers dispersed throughout the whole genome following standard protocols [3].

Five candidate genes were chosen according to their role in trichothecene biosynthesis and/or aggressiveness (*TRI1, TRI5, TRI6, TRI10*, and *TRI14*). Two other, yet uncharacterized candidate genes in *Fusarium *spp. (*Erf2, MetAP1*) were tested for a possible association with aggressiveness. Nucleotide sequences of these genes were imported from *Fusarium graminearum *database FGDB [37]. The selected genes were (Table 3): *TRI1 *(FGSG_00071), *TRI5 *(FGSG_03538), *TRI6 *(FGSG_16251), *TRI10 *(FGSG_03538), *TRI14 *(FGSG_03543), *MetAP1 *(FGSG_01397), and *Erf2 *(FGSG_08531). Specific primers were designed (see Additional file 2) to amplify parts of these genes using the software Primer Premier 4.0 (Premier Biosoft International, CA, U.S.A.).

**Table 3:** Name of tested genes, chromosomal localization, primer sequence and expected amplified DNA products

**No**.	Gene ID^a^	Chromosome		Sequences of the primers	Ta (°C)	Expected products (bp)	Gene size (bp)
1	*TRI1 *(FGSG_00071)	I	F	CACCAGTTTGCAGGATGT	53.2	850	1980
			R	AATGGGAGTGATTAGTTCG			
2	*TRI5 *(FGSG_03537)	II	F	TGGCGGATCTATCTATTCA	57.1	750	1129
			R	CTTCTTGGCGTCCTCTGT			
3	*TRI6 *(FGSG_1625)	II	F	ATGGAGGCCGAATCTCAC	54.4	600	673
			R	CCACCCTGCTAAAGACCCT			
4	*TRI10 *a (FGSG_03538)	II	F	ATTTCCCAAAGCCTAGACAA	54.2	700	1264
			R	GGCCGTAATCTTCAAATGGT			
5	*TRI10 *b (FGSG_03538)	II	F	GCACCATTTGAAGATTACGG	54.2	550	
			R	CGTCAAGTCTTCCCATCTCA			
6	*TRI14 *(FGSG_03543)	II	F	AGCCCGAACCCGCTACAT	57.3	750	1116
			R	CGAACCTGCTGCTCTTAC			
7	*MetAP1 *(FGSG_01397)	I	F	AACGATGCCGGATCGTCA	55.3	800	1403
			R	TTGCCAAACTCTCGGATCAA			
8	*Erf2 *(FGSG_08531)	IV	F	AGGCATCTTTGTTGTTGTG	52.4	750	2040
			R	TTGGTAATACGTGGGTTGT			

^a ^The given gene ID is the entry number of the MIPS *F. graminearum *genome database (FGDB; Wong et al. 2011)

Polymerase chain reaction (PCR) was performed using the designed primers related to each gene separately following standard protocols, however, with a different annealing temperature for each gene (Table 3). Purification of PCR products was performed by precipitating the DNA with 10% (v/v) of 3 M Sodium Acetate and 25% (v/v) of absolute cold ethanol overnight. Precipitated DNA was cleaned twice with 70% ethanol and finally diluted in 10 μl ddH_2_O. Purified PCR products were sequenced (QIAGEN^® ^Sequencing Services, Hilden, Germany). The sequence was performed once for each isolate, hence the alignment was mostly identical (the sequence was repeated in case it fail to align to the other sequence or in case of high noise in the sequence). Expected sizes of PCR products were obtained for all tested genes. All sequences of *TRI6 *and *TRI14 *were located in the coding region of the genes (exon, see Additional file 1). Sequences of the genes *TRI5, MetAP1, Erf2*, and *TRI10 *(two parts) were stretched over two exons. Sequences of genes were obtained from right to left sides of PCR products, just genes *MetAP1 *and *Erf2 *were obtained from left to right side. Sequence of *TRI1 *was located over four exons according to the reference sequence. Number of identified SNPs was high over all sequenced regions of candidate genes (Table 1). The sequences were aligned (according to the initial matrix published in FGDB) using CLC sequence viewer 6.3 (CLC-bio, Aarhus, Denmark) to identify single nucleotide polymorphisms (SNPs) among the 77 isolates.

### Phenotypic data analyses

The following linear mixed model was used to estimate variance components: *y_ijn _*= *μ*+*Iso_i_*+*Env_j_*+(*Iso *× *Env*)*_ij _*+*Rep_n_*+*e_ijn_*, where *μ *is the population mean, *Iso_i _*the genetic effect of the *i*th isolate, *Env_j _*the effect of the *j*th environment, *Iso *× *Env *the isolate times environment interaction effect, *Rep_n _*the effect of the *n*th replication, and *e_ijn _*the residual error. Variance components were determined by the restricted maximum likelihood (REML) method using the software ASReml 2.0 (VSN International Ltd, Hemel Hempstead, U.K.). Significance for variance component estimates was tested by model comparison with likelihood ratio tests where the halfed *P *values were used as an approximation [38]. Heritability (*h^2^*) on an entry-mean basis was estimated as the ratio of genotypic to phenotypic variance according to Melchinger et al. [39]. Furthermore, genotypes were regarded as fixed effects and best linear unbiased estimates (BLUEs) were determined for all isolates and traits.

Genetic relatedness among the 77 isolates was determined by applying principal coordinate analysis (PCoA) [40] based on the modified Rogers' distances of the isolates [41]. Linkage disequilibrium (LD) between the selected SNPs was assessed by the LD measure *r^2 ^*[42] and significance of LD was tested with Fisher's exact tests [43]. LD and PCoA computations were performed with the software package Plabsoft [44].

### Association analysis

A two-step association approach was applied in this study and the BLUEs per environment were used as input for the association analysis. The linear mixed model for the association approach was: *y_ijn _*= *μ*+*a_p_*+*Iso_i_*+*Env_j_*+*e_ijp_*, where *a_p _*is the effect of allele *p*. The allele effect *a_p _*was modeled as fixed effect whereas *Iso_i _*and *Env_j _*were regarded as random effects. We assumed that the variance of the random genetic effect was Var(*g*) = 2Kσ^2^*_g_*, where σ^2^*_g _*refers to the genetic variance estimated by REML and K was a 77 × 77 matrix of kinship coefficients that define the degree of genetic covariance between all pairs of entries. We followed the suggestion of Bernardo [45] and calculated the kinship coefficient K*_ij _*between isolates *i *and *j *on the basis of the SSR marker data as *K_ij _*= 1+(*S_ij_*-1)/(1-*T_ij_*), where S*_ij _*is the proportion of marker loci with shared variants between isolates *i *and *j*, and T*_ij _*is the average probability that a variant from one isolate *i *and a variant from one isolate *j *are alike in state, given that they are not identical by descent. The coefficient T*_ij _*was estimated separately for each gene and trait using a REML method setting negative kinship values between isolates to zero. SNPs with allele frequencies < 0.1 were not considered in the association analysis or LD estimation. The obtained optimum T values were for DON content: *TRI1 *(0.025), *TRI5 *(0.575), *TRI10 *(0.375), *MetAP1 *(0.300), and *Erf2 *(0.400). For mean FHB rating: *TRI1 *(0.600), *TRI5 *(0.150), *TRI10 *(0.200), *MetAP1 *(0.075), and *Erf2 *(0.275). For the detection of main effects of the candidate gene SNPs, these were fitted as fixed effects in the mixed model and their significance was tested by a Wald *F *test. Based on the Wald *F *statistic, we performed tests for the presence of marker-phenotype associations with a significant (*P *< 0.05) effect on DON content and mean FHB rating applying the Bonferroni-Holm procedure [22] to correct for multiple testing.

The proportion of genotypic variance (*p_G_*) explained by the detected SNP was calculated by fitting each SNP in a linear model to obtain *R^2^_adj_*. The ratio *p_G _*= *R^2^_adj_*/*h^2 ^*yielded the proportion of genotypic variance [46]. In the case of *MetAP1*, where two SNPs were detected for mean FHB rating, both were simultaneously fitted in the linear model in the order of their *P *values to correct for collinearity. Haplotype analyses were performed with the same procedure to test the association with both phenotypic traits (Additional file 2: Table S1). We applied the same threshold level like for SNP analyses. All mixed model calculations were performed using the software ASReml 2.0 (VSN International Ltd, Hemel Hempstead, U.K.).

## Competing interests

The authors declare that they have no competing interests.

## Authors' contributions

FT carried out the phenotypic and molecular analyses, performed parts of the statistical analyses and drafted the manuscript. TW performed parts of the statistical analyses and helped to draft the manuscript, JCR edited the manuscript, HKP† participated in the design of the study and supported the technical realization. TM participated in the design of the field study and helped to draft the manuscript. All authors read and approved the final manuscript.

## Supplementary Material

Additional file 1 **Genomic organizations of the seven candidate genes with exons in black color**. The sequenced region is shown as a green bar above each gene. Click here for file

Additional file 2 **Table S1**. Haplotype analysis of the genes *MetAP1 and TRI1*. Click here for file
