# Supplementary material for: Association of single nucleotide polymorphic sites in candidate genes with aggressiveness and deoxynivalenol production in Fusarium graminearum causing wheat head blight
Source: BMC Genet. 2012 Mar 12;13:14. doi: 10.1186/1471-2156-13-14 (PMC3361471; doi:10.1186/1471-2156-13-14)
Supplement: Additional file 2 — Table S1. Haplotype analysis of the genes MetAP1 and TRI1. [file 1471-2156-13-14-S2.DOC]

**Supplemental Table 1. Haplotype analysis of the genes*MetAP1 and TRI1^a^*.**

| Gene | No. of haplotypes | Haplotype counts | | | Mean FHB rating (P-value) | | DON production (P-value) | |
| --- | --- | --- | --- | --- | --- | --- | --- | --- |
|  |  | Min | Max | |  |  |  |  |
| *MetAP1* | 35 | 1 | | 17 | | **0,0006** | | 0,1878 |
| *TRI1* | 3 | 6 | | 30 | | 0,7325 | | **0,0024** |

^a^ Given are the number of haplotypes per gene, the range of the number of isolates within each haplotype (haplotype count).
